# Rhizosphere Soil Fungal Community Patterns and Drivers in Chinese Fir Plantations Across Developmental Stages

**DOI:** 10.3390/microorganisms14092078

**Published:** 2026-09-17

**Authors:** Hui Zhang, Chunmei Xie, Hui Wang, Daoqiang Lu, Lin Qiu, Guannan Ma, Yuefeng Li, Lisha Fang, Yun Zhang

**Affiliations:** 1 College of Forestry, Xinyang Agriculture and Forestry University, Xinyang Academy of Ecological Research, Xinyang 464000, China; hui_2013wang001@163.com (H.Z.);; 2 Henan Dabieshan National Field Observation and Research Station of Forest Ecosystem, Zhengzhou 450046, China; 3 College of Forestry, Jiangxi Agricultural University, Nanchang 330045, China; 4 Jigongshan National Nature Reserve, Xinyang 464039, China; 5 Xinyang Forestry Science Research Institute, Xinyang 464000, China

**Keywords:** fungal community assembly, plant–soil–microbial interaction, root exudates, forest age

## Abstract

The composition, richness, and diversity of rhizosphere fungal communities play significant roles in plant development in Chinese fir plantations. Understanding their structure and assembly, along with their relationship to soil physicochemical properties and root traits, is essential for maximizing the benefits of fungal symbionts for Chinese fir trees. Hence, fungal communities in two soil layers (0–20 cm and 20–40 cm) at four plantation developmental stages were analyzed using internal transcribed spacer rRNA gene sequencing. Soil physicochemical properties and root traits were examined to determine the main fungal community drivers. Our results revealed that fungal communities were significantly influenced by stand developmental stage, rhizosphere, and soil depth. Fungal biomass was highest at the young stand stage, whereas the α-diversity and evenness of the surface rhizosphere soil fungi remained stable across stages. With increasing stand age, the fungal community structure shifted from aggregated to dispersed. Co-occurrence network analysis demonstrated that stand development promoted network connectivity and modularity, with tighter local associations and distinct core functional consortia formed in the mature and over-mature stages. The proportion of positive links and positive cohesion increased along the stand developmental gradient, indicating a shift from competitive to cooperative interspecific interactions. Ascomycota and Basidiomycota consistently dominated soil fungal communities regardless of stand age, whereas Mortierellomycota and Chytridiomycota displayed sensitivity to stand development despite their low relative abundance. Soil physicochemical properties (total carbon, potassium, dissolved organic carbon, and phosphorus) were the primary drivers of rhizosphere fungal community dynamics and regulated fungal assembly by controlling soil resource availability. Additionally, the structural configuration of the fungal community was found to be intricately linked to root biomass and morphological traits.

## 1. Introduction

Microorganisms are essential for maintaining soil health and ecosystem equilibrium. They hence play an indispensable role in the growth and survival of plants and other dependent organisms. Fungi represent a ubiquitous and functionally diverse group that drives critical ecological processes, including nutrient cycling, carbon sequestration, soil formation, and the establishment of symbiotic relationships with plants [1]. As a dominant component of soil microbial biomass, fungal communities are often more sensitive indicators of soil quality and ecosystem health than bulk physicochemical properties. Functionally, saprotrophic fungi drive the decomposition of complex organic substrates into simple inorganic constituents that are subsequently recycled and utilized by plants and other organisms [2], whereas mycorrhizal fungi establish mutualistic associations with plant roots, enhancing the uptake of water and essential nutrients while concurrently bolstering host tolerance to abiotic and biotic stressors [3]. In parallel, endophytic fungi further modulate plant–soil feedbacks by reshaping root exudation patterns and altering rhizosphere chemistry, thereby contributing to plant adaptability and resilience [4]. In contrast, some fungal taxa adopt pathogenic lifestyles, suppressing host growth and reproduction through tissue colonization [5]. Consequently, a comprehensive characterization of fungal community structure, diversity, and functional guild composition is paramount for evaluating the status of forest soil health and guiding sustainable ecosystem management.

The rhizosphere is defined as a narrow zone of soil extending only a few millimeters from the root surface, where dynamic root–soil–microbe interactions create a distinct biogeochemical environment. Continually enriched with labile carbon and low-molecular-weight root exudates, this compartment constitutes the most metabolically active hotspot in terrestrial ecosystems, supporting rates of nutrient cycling and microbial activity that far exceed those of bulk soil [6]. This dynamic interface functions as a central hub for substrate flux and bidirectional plant–microbe interactions, through which the rhizosphere microbiome directly modulates plant growth, development, and community dynamics [7,8]. The specialized microbial assemblages also enhance plant stress tolerance and play a pivotal role in shaping plant community structures [8]. In forest ecosystems, the succession of rhizosphere fungal communities follows a clear ontogenetic trajectory across stand developmental stages. As plantations transition from young to over-mature stages, community composition shifts directionally, with early stages often dominated by symbiotic ectomycorrhizal taxa and later stages favoring the proliferation of saprotrophic fungi [9,10,11]. Although stand age is a key driver of rhizosphere microbial community assembly, the mechanisms underlying its regulation of fungal community structure and functional shifts under field conditions remain poorly understood.

As plantations progress through successive developmental stages, two interconnected trajectories emerge. At the individual level, tree ontogeny drives shifts in root functional traits and exudate profiles [12], whereas at the stand level, structural attributes—including canopy architecture, vertical stratification, and understory composition—undergo substantial reorganization due to both management practices and natural processes [13,14]. These concomitant aboveground changes cascade into the belowground environment by modulating the forest floor microclimate, altering the quantity and recalcitrance of litter inputs, and reshaping soil physicochemical properties [9,11,15]. This hierarchical cascade of age-dependent modifications, ranging from individual tree physiology to stand-level structural dynamics, introduces considerable variability in the drivers of fungal community succession. A growing body of evidence has documented pronounced shifts in the composition and diversity of rhizosphere microbial communities across the developmental gradients of stands in Chinese fir plantations. Notably, both bacterial and fungal assemblages exhibit significant successional dynamics from the young to over-mature stages, reflecting coordinated changes in soil biotic and abiotic conditions [16,17]. Similar patterns have been reported in other forest systems, where the fungal community structure varies significantly across successional stages [18], and distinct differences in root-associated fungal assemblages—particularly ectomycorrhizal and ericoid mycorrhizal fungi—have been observed between young and mature stands [19]. These successional shifts in fungal communities are closely associated with shifts in plant nutrient acquisition strategies and tissue stoichiometry. As Chinese fir plantations mature, the progressive accumulation of recalcitrant litter and the depletion of readily available soil nutrients drive a functional transition from symbiotic to saprotrophic fungal guilds [11,17]. Concurrently, the stoichiometric balances of carbon (C), nitrogen (N), and phosphorus (P) in plant tissues and soil organic matter undergo marked changes, reflecting age-dependent variations in nutrient demand and cycling efficiency [20,21,22]. Collectively, these coupled aboveground–belowground dynamics indicate that stand age is not merely a temporal variable but a composite driver that integrates plant physiological status, edaphic conditions, and microbial community assembly. Therefore, elucidating the successional trajectories of soil microbial communities and their environmental determinants is essential for understanding the ecological mechanisms underlying soil degradation and productivity decline in Chinese fir plantations across successive rotations.

*Cunninghamia lanceolata* (Lamb.) Hook is a native tree species endemic to China that is valued for its rapid growth, high timber yield, and versatile wood utilization, which collectively underpin its significant role in regional economic development. As one of the most extensively cultivated conifers in subtropical Asia, Chinese fir plantations cover approximately 9.9 million hectares in China, accounting for approximately 25% of the nation’s total planted forest area and making a substantial contribution to global plantation forestry [23]. However, the continuous expansion of Chinese fir monoculture plantations, often managed under short rotations and successive replanting, has raised considerable concerns regarding the progressive decline in soil fertility and diminished long-term stand productivity [17,24]. Recent studies have indicated that root-associated microbial communities are closely correlated with host growth traits in Chinese fir, with fungal functional guilds undergoing marked successional shifts from symbiotic to saprotrophic dominance as stands mature [10,11,25]. Despite these advances, the underlying mechanisms remain unclear. In particular, the relative contributions of soil physicochemical properties and root functional traits in shaping rhizosphere fungal community assembly across different stand developmental stages have yet to be fully resolved.

To address this knowledge gap, we selected C. lanceolata plantations of four stand ages (6, 14, 23, and 36 years) to represent the key developmental stages across a typical rotation cycle. This study aimed to: (1) investigate the diversity, composition, and assembly patterns of rhizosphere fungal communities across the developmental stage gradient, and (2) quantify the relative contributions of soil physicochemical properties and root functional traits in shaping fungal community dynamics. By integrating plant traits, soil properties, and microbial community data, this study sought to identify the key drivers of rhizosphere fungal succession and provide a mechanistic framework for devising forest management strategies that enhance nutrient sustainability and long-term productivity in Chinese fir plantations.

## 2. Materials and Methods

### 2.1. Study Site Description

The study site was located at the Xinkou Experimental Forestry Center (26°11′30″ N, 117°26′00″ E) of Fujian Agriculture and Forestry University in Sanming, Fujian Province, China. The climate is a typical subtropical monsoon, with the mean annual rainfall and mean annual temperature of the study area being 1700 mm and 19.6 °C, respectively [26].

In January 2019, 12 plots were established at four stand developmental stages, with three southward quadrangular plots (20 m × 20 m) in each stage. Each stand had two downhill plots and one uphill plot to reduce the impact of slope location on soil microorganisms. Soil samples were taken from Chinese fir plantations with trees aged 6, 14, 23, and 36 years, which reflected the typical developmental stages of Chinese fir: young, middle-aged, mature, and over-mature [27]. These stands, all in their first rotation, were established after clear-cutting and one slash-burning of natural forests dominated by *Pinus massoniana* (Lamb.) during the site preparation. The reforestation process involved standard procedures, such as biannual weeding and nursery operations for three years after reforestation. Regarding stand management, two thinning operations were scheduled during the rotation cycle, with the first thinning conducted approximately 8–12 years after establishment and the second 15–20 years after establishment. The corresponding thinning intensities ranged from 25 to 35% and 15–20%, respectively, relative to the initial stand density. Specifically, the 14-year-old stand was thinned once in 2015 at an intensity of 30%, the 23-year-old stand underwent two thinning events with thinning intensities of 28% and 18%, and the 36-year-old stand underwent two thinning operations at intensities of 25% and 20%, The age and background information of the Chinese fir plantations were obtained through communication with the local forestry authorities. The four selected stands had similar afforestation backgrounds, management practices, forest histories, and origins, ensuring that the amount and quality of organic matter entering the soil layer remained consistently comparable over the long run. The maximum linear distance between any two stands did not exceed 10 km [11]. This relatively short spatial distance was selected to minimize large-scale environmental heterogeneity and enhance comparability among the plantations at different developmental stages. According to a 1999 soil survey, the selected stands were classified as Ultisols, which are widely distributed in southern China and typically characterized by strong acidity and intensive weathering. In this study, soil pH values ranged from 3.95 to 4.30 across all stand ages and soil depths (Table S1), confirming the acidic nature of the studied soils. Despite the differences in stand locations, various factors such as geographical distance, forest history, afforestation methods, bedrock, slope direction, and topographic slope were carefully considered when selecting the test stands to ensure homogeneity in initial conditions, including soil, climate, and vegetation. Consequently, the succession and driving factors of the soil microbial community in the Chinese fir stand are predominantly influenced by the developmental stage of the plantation.

### 2.2. Plant and Soil Sampling and Treatment

In each block, the position was determined by the average diameter at breast height of the standard trees. The root systems of the Chinese fir trees were excavated at depths of 0–20 cm and 20–40 cm in a 1 m radius area, with a sector angle of 60° in the uphill and downhill directions, starting from the tree base. The side roots of Chinese fir trees were exposed and separated from the roots of other tree species within the designated sampling area. Chinese fir roots with adhering soil were carefully collected because of the high cohesiveness of southern red loam. The loosely attached soil particles were removed by gentle shaking, and the soil layer within approximately 5 mm of the root surface was manually separated and defined as the rhizosphere soil. This operational definition was based on previous studies that considered the soil layer closely adhering to the roots as the rhizosphere soil [7]. The roots and rhizosphere soil were collected and stored with ice packs before being sent to the laboratory for analysis.

The root system was cleaned using a sterilized brush, and the inter-root soil of the plant in the same layer was thoroughly mixed and divided into three parts. One portion of the soil was dried to assess its physical and chemical properties, while another portion was refrigerated at 4 °C for measuring the soil dissolved organic carbon (DOC) and inorganic nitrogen. The remaining part was stored in an ultra-low temperature refrigerator at −80 °C for DNA extraction. After rinsing and cleaning, the fir roots were classified into first-, second-, and third-order roots using Strahler’s stream-ordering system [28]. Because of the serious damage to the collected roots, it was not possible to trace a sufficient number of class 3 root segments; therefore, we only analyzed class 1 and class 2 roots. Images of all roots were obtained using a Canadian digitizing scanner (STD1600 Epson, Seiko Epson Corporation, Los Alamitos, CA, USA). The WinRHizo Root Analysis System (Pro 2007d, Regent, Quebec, QC, Canada) was used to quantitatively evaluate root length, surface area, volume, and diameter. Specific root length and tissue density were determined from root morphology and biomass data. Subsequently, the roots were dried at 65 °C, weighed for biomass determination, and crushed to analyze phosphorus content.

### 2.3. Analysis of Soil Physical and Chemical Properties

Soil pH was measured at a soil-to-water ratio of 1:2.5, and total nitrogen (TN) and carbon were analyzed using an elemental analyzer (VARIO MAX CN, Elementar, Hanau, Germany). The soil was digested with H_2_SO_4_ to determine the total P content. The available phosphorus (AP) in the soils was extracted using 0.03 M NH_4_F–0.025 M HCl, followed by colorimetric determination using the molybdenum-antimony method [29]. Metal elements in the soil, potassium (K), calcium (Ca), and magnesium (Mg), were analyzed using an ICP-OES instrument (OPTIMA 8000, PerkinElmer, Waltham, MA, USA) following digestion with HF-HClO_4_. The water content of the non-irrigated soil was determined using the drying method, whereas the soil particle composition was analyzed using the burette method. The DOC in the solution was measured using a total organic carbon analyzer (TOC-L, Shimadzu Corp., Kyoto, Japan). To determine the nitrate nitrogen (NN) and ammonium nitrogen (AN) concentrations, a solution was prepared by combining 3.0 g of fresh soil and 30 mL of a 2.0 mol L^−1^ KCl solution. The mixture was then shaken at 250 rpm for 1 h and centrifuged at 9000 rpm for 10 min at 25 °C. The supernatant was collected and analyzed using a flow analyzer (AA3, SEAL Analytical Ltd., Norderstedt, Germany).

### 2.4. Fluorescence Quantitative Polymerase Chain Reaction (qPCR) Analysis of Rhizosphere Fungal Abundance

The relative abundance of fungal internal transcribed spacer (ITS) gene copies in soil DNA was measured using fluorescent real-time polymerase chain reaction (PCR; Applied Biosystems, Foster City, CA, USA). The fungal ITS region was amplified using the primer pair ITS1F (5′-CTTGGTCATTTAGAGGAAGTAA-3′) and ITS2 (5′-TGCGTTCTTCATCGATGC-3′). The reaction mixture contained 2 μL of DNA template, 10 μL of 2× Taq Master Mix (Vazyme, Nanjing, China), 0.5 μL each of 10 μM forward and reverse primers, and ddH_2_O. The qPCR conditions were pre-denaturation at 94 °C for 3 min, followed by 30 cycles of denaturation at 94 °C for 30 s, annealing at 55 °C for 30 s, and extension at 72 °C for 30 s. A plasmid standard was then created with a 10-fold dilution of 10^1^–10^5^ copy numbers. Three replicates of each sample were averaged to obtain the results, which were expressed as the number of DNA copies per unit dry soil mass (copies g^−1^ dry soil) [28].

### 2.5. Extraction of Total DNA of Rhizosphere Fungal Communities

For each soil sample, 500 mg was weighed, and total soil DNA was extracted using the E.Z.N.A.^®^ Soil DNA Extraction Kit (Omega Bio-tek, Norcross, GA, USA). Three replicates were created for each sample, and the extraction method was performed according to the manufacturer’s instructions. All extracted DNA was analyzed using 1% agarose gel electrophoresis, and sample DNA quality, concentration, and fragment length were assessed with a NanoDrop 2000 spectrophotometer (Thermo Fisher Scientific, Wilmington, DE, USA) to ensure that the samples met the requirements for subsequent analysis.

The reverse primer ITS2 (5′ TGC GTT CTT CAT CGA TGC 3′) was used to amplify the ITS1 region of fungal ribosomal DNA. For each DNA sample, a triplicate reaction of 25 μL was prepared, containing 30 ng of template DNA, 1 μL of the forward fusion primer (5 μM), 1 μL of the reverse fusion primer (5 μM), 3 μL of 2× Taq Plus Master Mix, 3 μL of bovine serum albumin (BSA, 2 ng μL^−1^, Merck, Kankakee, IL, USA) and 7.5 μL of ddH_2_O. The thermal cycling conditions for the fungal ITS region were set to 5 min at 94 °C; 34 cycles of 30 s at 94 °C, 30 s at 55 °C, and 60 s at 72 °C; and 7 min of extension at 72 °C. Replicate amplicons were pooled and subjected to high-throughput sequencing by Beijing Allwegene Tech, Ltd. (Beijing, China) using an Illumina MiSeq PE300 sequencing platform (Illumina, Inc., San Diego, CA, USA).

To ensure accuracy, sequences with lengths shorter than 200 bp and Phred quality scores lower than 20 were removed from the raw data. Non-specific amplified sequences and chimeras were then removed, and sequences with greater than 97% similarity were clustered into operational taxonomic units (OTUs) using UPARSE software (v2.7.1). BLAST+ 2.16.0 was used to classify all sequences into species using the GenBank Release 272.0 non-redundant nucleotide database. Alpha diversity metrics such as the Chao1 richness index, number of observed species, phylogenetic diversity index (PD whole tree), and Shannon-Wiener diversity index were then calculated using QIIME (v1.8.0).

### 2.6. Statistical Analysis

Microsoft Excel 365 and R (version 4.0.0) were used for data sorting, statistical analysis, and plotting. Two-way analysis of variance (ANOVA) was used to examine the effects of forest development stages and soil depth on the abundance of fungal ITS gene copies and diversity indices (OTU richness and Shannon evenness). Microbial gene abundance and the relative abundance of fungal phyla at various soil layers and forest development stages were analyzed using one-way ANOVA, with means assessed using Tukey’s honest significant difference test at the 5% level.

Because of the challenges in technical and statistical reproducibility associated with low-abundance OTUs, only OTUs with an abundance greater than 0.5% in more than five samples were used to determine differences in abundance. The normality and homogeneity of variance were evaluated using the Shapiro–Wilk and Bartlett tests prior to conducting the ANOVA. When the assumption of a constant variance was violated, the data were log-transformed or calculated as square roots to satisfy the assumptions of ANOVA.

Using the Hellinger-transformed OTU relative abundance, the Bray–Curtis method was employed to calculate the distance matrix of the fungal communities. Subsequently, non-metric multidimensional scaling (NMDS) based on the Bray–Curtis distance was used to explore the differences in fungal OTUs at different soil depths across various developmental stages of *Cunninghamia lanceolata* (metaMDS function). A relative abundance heatmap of the fungal functional groups across the four developmental stages was created using the pheatmap package. A Spearman correlation matrix between the root traits, soil properties, and fungal functional groups was computed using the corr.test function in the psych package, and the correlation heatmap was visualized using ggplot2 3.5.2.

**Figure 1:**
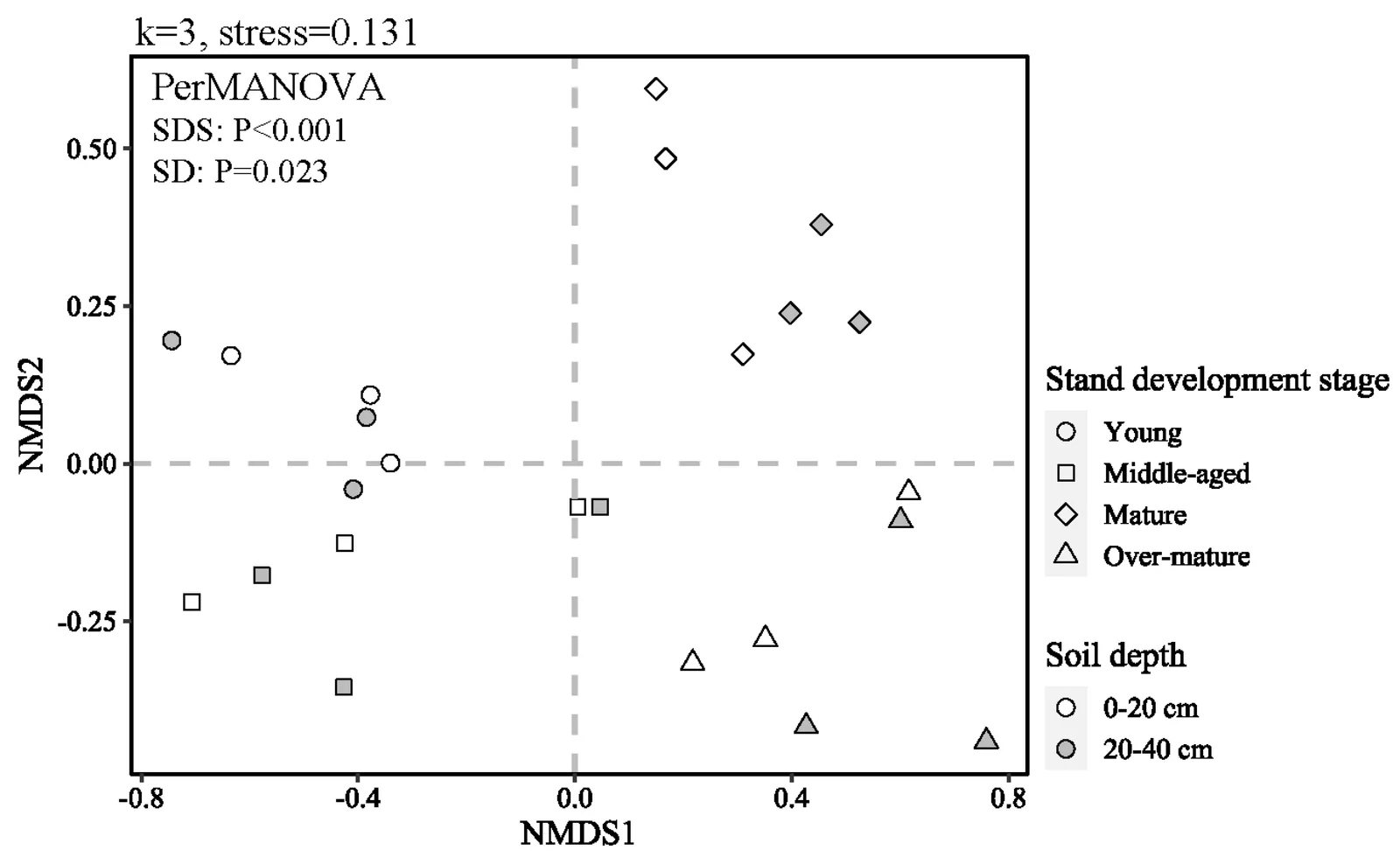
NMDS ordination of fungal OTU matrices based on Hellinger-transformed Bray–Curtis distances at depths of 0–20 cm and 20–40 cm during different stand developmental stages of Chinese fir plantations. The effects of stand development stage (SDS), soil depth (D) and their interaction (SDS × D) on the composition structure of the fungal community were assessed using PerMANOVA.

A permutational ANOVA with 999 permutations was conducted to test for the significant effects of forest development stages and soil depth on microbial communities using the adonis function. Pairwise comparisons used the Holm correction for multiple tests, along with a custom function to compare microbial communities between different forest stands. Variance partitioning analysis (VPA; varpart function) was performed using Hellinger-transformed OTU abundance data as the response variable to quantify the relative contributions of soil and root traits to variations in fungal community structure (Table 1 and Table S1). The soil and root trait explanatory sets were fitted using a fungal abundance matrix (rda function). The importance of the variables in the explanatory model was determined through stepwise selection using the Akaike information criterion to remove non-significant variables from each explanatory set. The variance partitioning of fungal communities under various environmental matrices (soil properties and root traits) was calculated using RsquareAdj and ANOVA. Additionally, selected VPA variables, including soil factors and root trait variables (as shown in Table 1 and Table S1), were subjected to a distance-based redundancy analysis (db-RDA; rda function). Variables with a variance inflation factor greater than 10 were excluded from the VPA to avoid multicollinearity due to multiple zeros. The significance of the global RDA model was evaluated using the cca function. To test the predictive significance of the selected variables in the fitted model, a Monte Carlo permutation test (999 permutations) was employed (envfit function). We further assessed the significance of the correlations between NMDS scores and soil and root traits using post hoc permutation correlations (envfit function), and the results are shown in Figure 1. The PerMANOVA, NMDS, VPA, and db-RDA were performed using the vegan package.

All analyses were performed using R (R 4.0.4; R Core Team), with statistical significance set at *p* < 0.05. All NMDS, RDA, VPA, and linear correlation results were visualized using the ggplot2 package.

## 3. Results

### 3.1. Rhizosphere Fungal Biomass and Alpha-Diversity in Chinese Fir Plantations at Different Developmental Stages

Stand developmental stage had a significant effect on the biomass of rhizosphere fungi in Chinese fir (*p* = 0.030; Table 1). Specifically, the fungal biomass for the young stage reached 69.23 ± 46.71 and 43.67 ± 25.92 in the different soil layers, which was significantly higher than that in the other three age categories. Overall, the Chao1 richness index, observed species count, PD whole tree index, and Shannon–Wiener diversity index of rhizosphere soil fungi in Chinese fir forests exhibited minimal variation across developmental stages, indicating that the evenness and diversity of rhizosphere fungi remained relatively stable. Soil depth had a significant effect on the Chao1 richness index of rhizosphere fungi (*p* = 0.002), species count (*p* = 0.003), and PD whole tree index (*p* = 0.005). Specifically, the Chao1 richness index, observed species, and PD whole tree index in the surface soil layer were all higher than those in the sub-surface layer.

### 3.2. Rhizosphere Fungi Community Structure in Chinese Fir Plantations at Different Developmental Stages

The NMDS ordination based on Bray–Curtis dissimilarity showed that the fungal community composition in the rhizosphere soil of Chinese fir plantations can be grouped into four distinct clusters (stress = 0.131, Figure 1). However, as the stand age increased, fungal communities became increasingly dispersed. Moreover, the fungal community was strongly influenced by developmental stage (PerMANOVA, *p* < 0.001), with significant differences in community composition between the 0–20 cm and 20–40 cm soil layers (PerMANOVA, *p* = 0.023).

There were notable differences between the distributions of the OTUs in the two soil layers. Specifically, 228 core fungal OTUs (13%) were detected in the 0–20 cm soil layer, whereas 189 core fungal OTUs (12%) were identified in the 20–40 cm soil layer (Figure S1). The number of shared fungal OTUs in the 0–20 cm soil layer was generally higher in all four stand age categories compared with the shared OTUs in the 20–40 cm soil layer. Additionally, OTU richness in the surface soil layer of mature forests and the deeper soil layer of young forests was relatively high, accounting for 13% and 15%, respectively. This observation indicates that fungal diversity is greater in both the mature and young stages.

### 3.3. Taxonomic Composition and Functional Guilds of Rhizosphere Fungi in Chinese Fir Plantations at Different Developmental Stages

**Figure 2:**
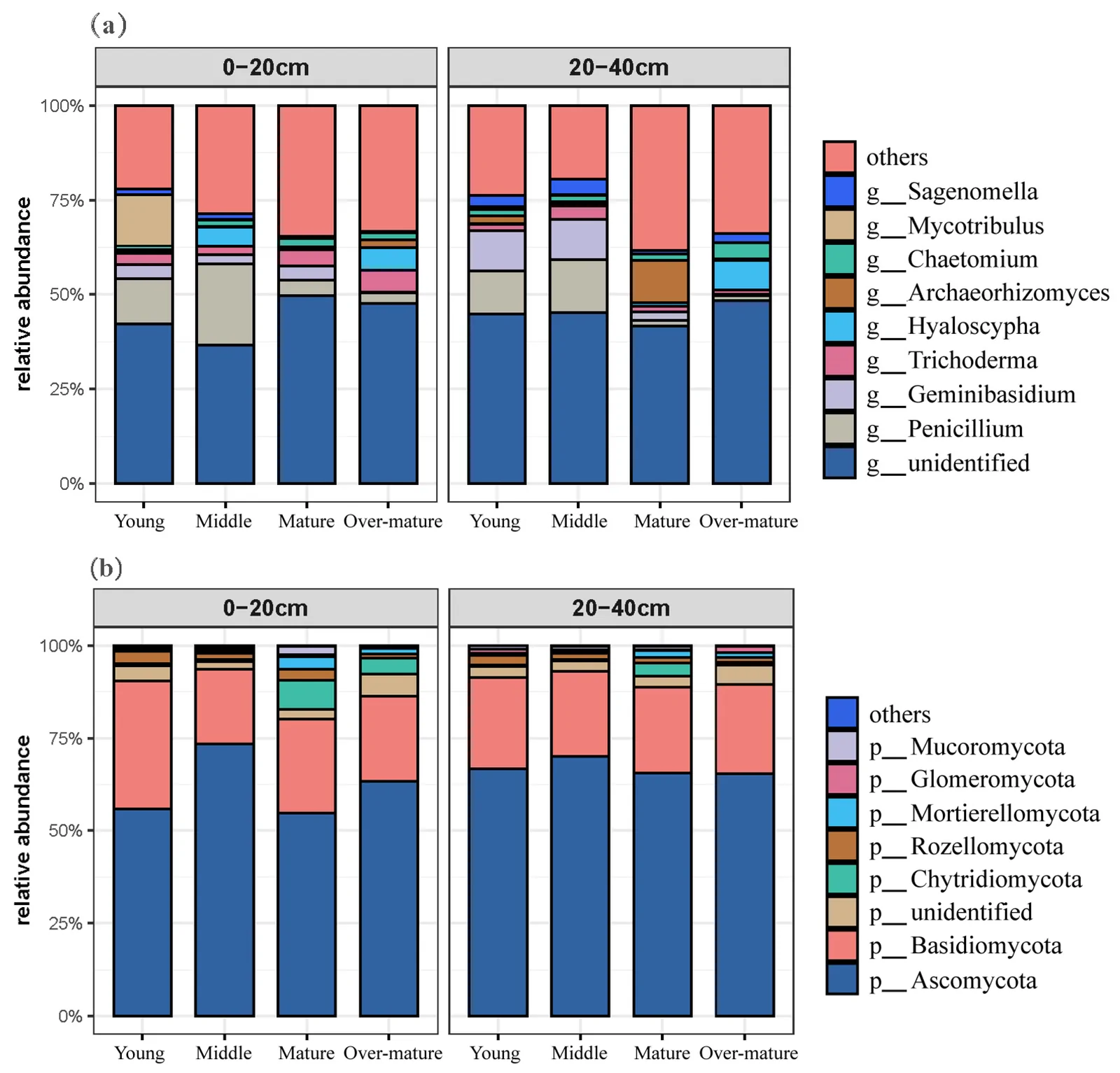
Average relative abundances of fungal phyla in the four stages of development. (**a**) Average relative abundances of fungal genera in 0–20 cm soil and 20–40 cm soil. (**b**) Average relative abundances of fungal phyla in 0–20 cm soil and 20–40 cm soil.

During the four developmental stages of the stands, the rhizosphere soil fungal community was predominantly composed of unknown genera (44.75%) and other genera (29%) (Figure 2a), neither of which was strongly influenced by stand development and soil depth. The genus *Penicillium* was more abundant in young and middle-aged forests than in mature and over-mature forests, suggesting that the developmental stage significantly influences this genus. Furthermore, the sub-surface layer showed a relatively high presence of *Geminibasidium* and *Sagenomella*, with percentages of 10.05% and 2.5%, respectively.

The soil fungal community at the phylum level was primarily composed of Ascomycota (64.36%) and Basidiomycota (24.77%). Specifically, the relative abundances of *Ascomycota* in the 0–20 cm and 20–40 cm layers were 61.82 ± 5.60% and 66.89 ± 33.18%, respectively (Figure 2b and Table S3). For Basidiomycota, the relative abundances in the 0–20 cm and 20–40 cm layers were 25.80 ± 4.95% and 23.74 ± 3.49%, respectively. The developmental stage of the stand had a minimal effect on these two dominant phyla, but it significantly affected the relatively less abundant Mortierellomycota and Chytridiomycota. The abundance of Mortierellomycota increased during stand development and peaked during the mature stage. As the stand developed, the number of unidentified phyla increased, peaking in over-mature stands. Moreover, the relative abundances of the various phyla were not significantly affected by soil depth.

**Figure 3:**
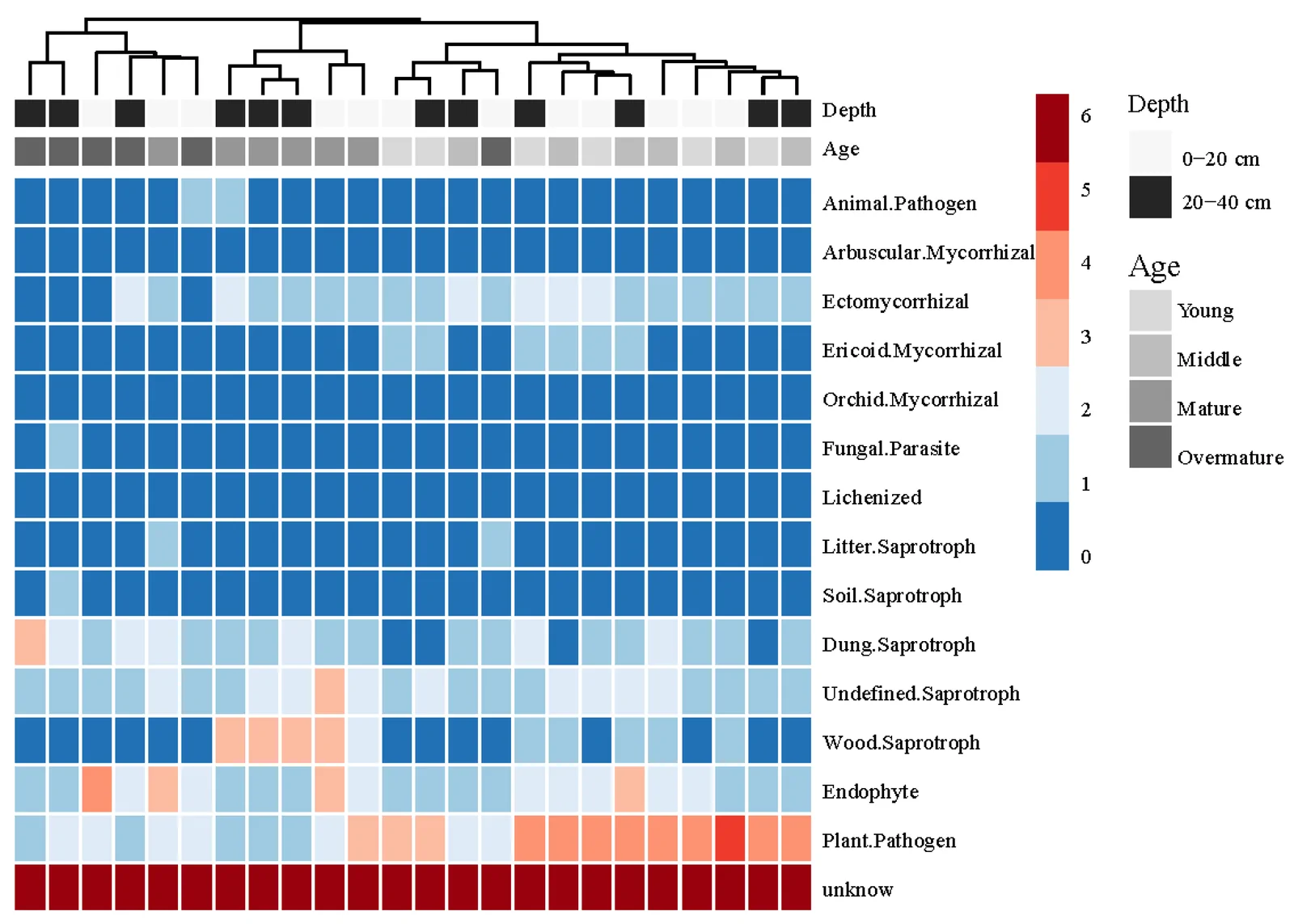
FUNGuild results for the relative abundance of fungi functional guild distribution in soils at 0–20 cm and 20–40 cm for the four stand development stages of Chinese fir plantations.

We used the FUNGuild database to annotate the fungi and identified three types of trophic modes: symbiotrophs, saprotrophs, and pathotrophs. The most abundant functional group was unknown fungi, followed by plant pathogens, endophytes, wood saprotrophs, undefined saprotrophs, dung saprotrophs, ectomycorrhizal fungi, and ericoid mycorrhizal fungi (Figure 3). Among them, plant pathogens (*p* < 0.001), ericoid mycorrhizal (*p* = 0.010), wood saprotrophs (*p* < 0.001) and undefined saprotrophs (*p* = 0.002) were significantly affected by the developmental stage of the stand (Table S3). Specifically, the abundances of plant pathogens and ericoid mycorrhizal decreased with increasing forest age. The abundances of wood saprotrophs and undefined saprotrophs were higher in the mature stage than in the other three stages.

Soil depth had a significant effect on undefined fungi (*p* = 0.005), with generally higher abundances of undefined saprotrophs in the surface soil than in the deeper layers. Both wood saprotrophs (*p* = 0.032) and unknown fungi (*p* = 0.025) were influenced by the combined effects of forest development stage and soil depth, with the highest abundances observed in the deeper soil of the mature stage.

### 3.4. Effects of Soil Chemical Properties and Root Traits on Changes in Rhizosphere Fungi Chinese Fir Plantations

**Figure 4:**
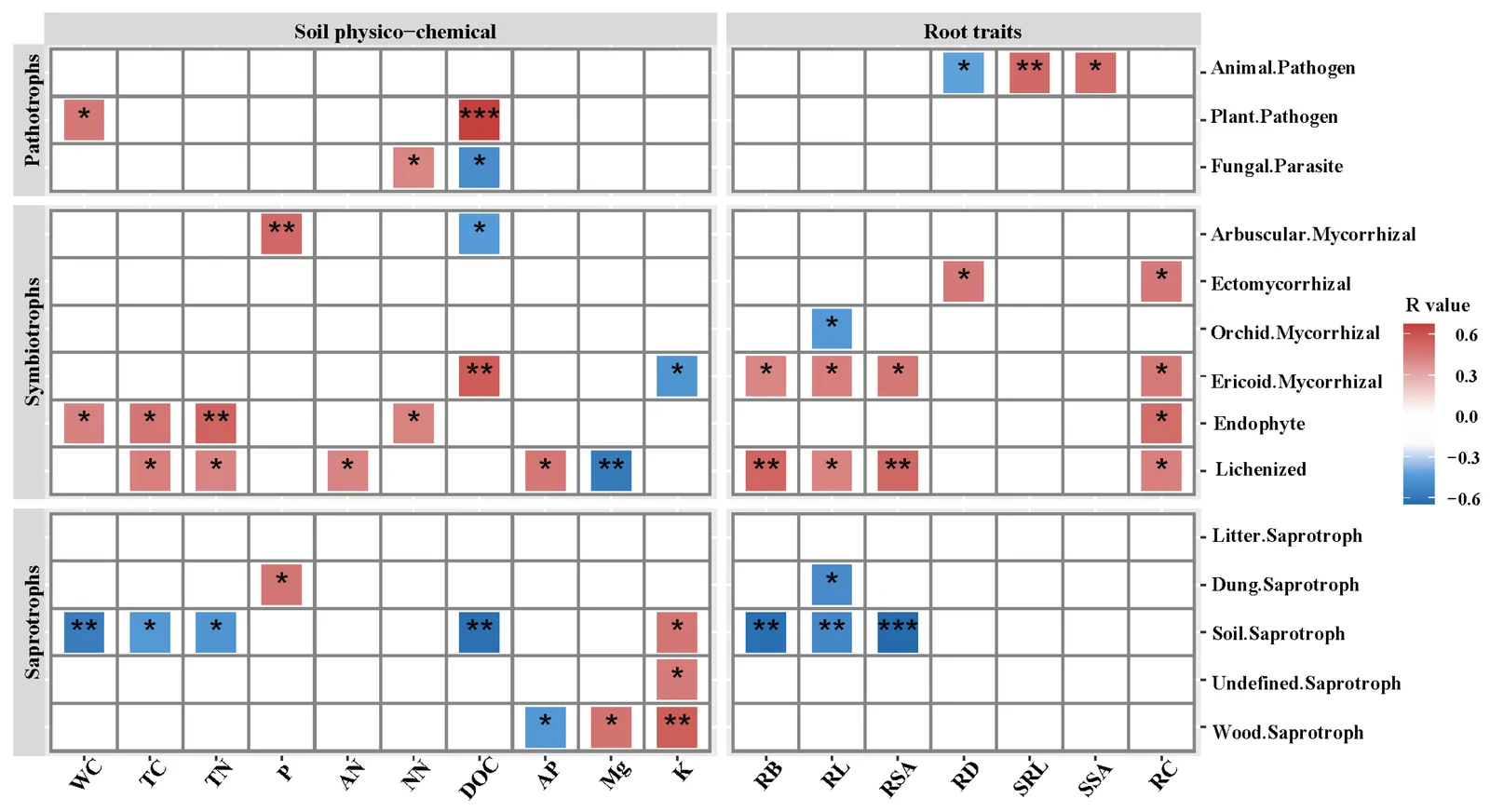
Correlation of different soil physicochemical properties and root characteristics to fungal functional groups (pathogenic fungal, symbiotic fungal, and saprotrophic fungal). Color indicates the correlation (R-value): red indicates positive correlation, blue indicates negative correlation, and significance is indicated by an asterisk (* *p* < 0.05, ** *p* < 0.01, *** *p* < 0.001).

The pathogenic fungi were influenced by soil properties and root traits. Specifically, animal pathogens were correlated solely with root traits, showing a positive correlation with specific root length (SRL) and specific root surface area (SSA; Figure 4). In contrast, the plant pathogens and fungal parasites were influenced by different factors. Plant pathogens were positively correlated with DOC and water content (WC), whereas fungal parasites were positively correlated with NN, but negatively correlated with DOC.

Symbiotic fungi exhibited distinct association patterns with soil properties and root traits. Specifically, the relative abundance of arbuscular mycorrhizal fungi was significantly and positively correlated with soil AP, whereas ectomycorrhizal fungi were positively associated with root architectural parameters, particularly root diameter (RD) and carbon content (RC). In contrast, the composition of endophytic fungal communities was predominantly correlated with edaphic variables, displaying significant positive relationships with soil WC, total carbon (TC), TN, and NN. Lichenized fungi responded to both soil physicochemical conditions and root morphological traits, and their abundance was positively correlated with root biomass (RB), root length (RL), root surface area (RSA), and RC, as well as with soil nutrient status indicators including TC, TN, AN, and AP. These correlative patterns underscore the differential environmental sensitivities of fungal functional guilds within the rhizosphere and root compartments.

Saprotrophic fungi showed negative correlations with soil properties and root traits. Specifically, they were negatively correlated with soil physicochemical properties such as WC, TC, TN, and DOC, as well as with root traits such as RB, RL, and root surface area (RSA). The physicochemical properties of soil influence wood. Saprotrophs were positively correlated with Mg and K, and negatively correlated with AP. K, P, and Mg were positively correlated with saprotrophic fungi, indicating that higher concentrations of these elements are associated with a greater abundance of saprotrophic fungi.

**Figure 5:**
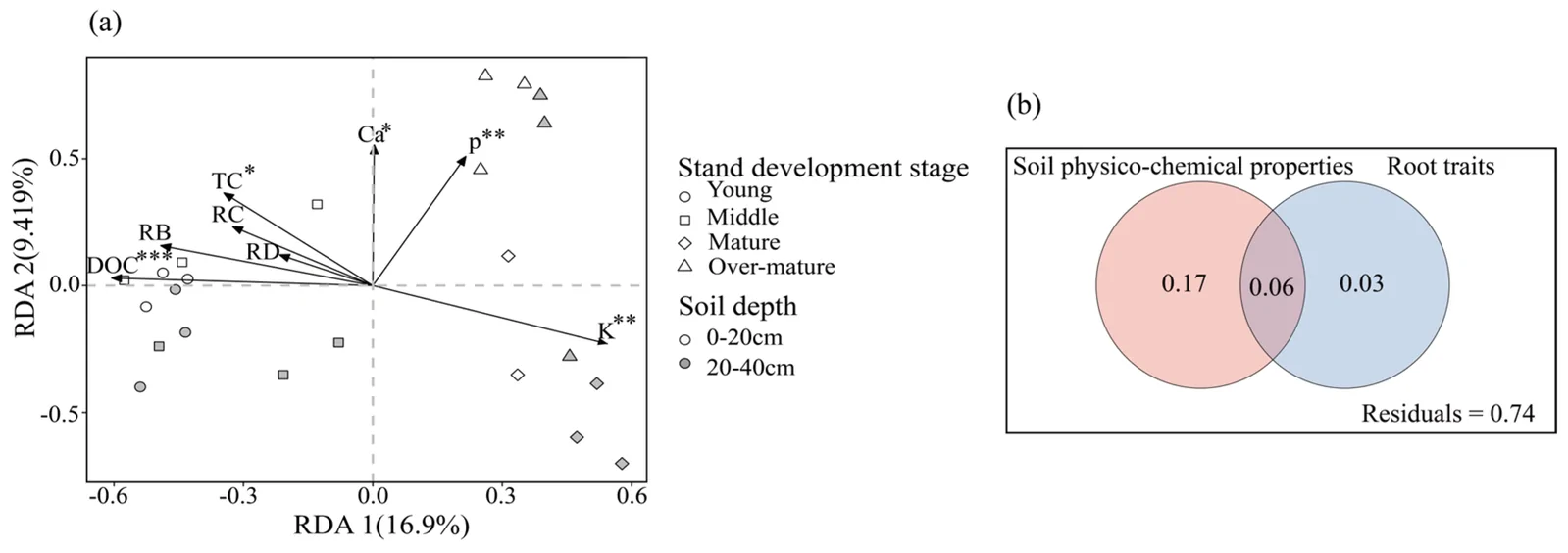
Relative contribution and influence of soil physicochemical properties and root traits on changes in the rhizosphere fungal community of Chinese fir forest. (**a**) Effects of soil physicochemical properties and root traits on soil fungal variation were analyzed using db-RDA. Arrow lengths indicate the degree of correlation between these variables and fungal community structure. (**b**) VPA results showing the relative contributions of soil physicochemical properties and root traits to variations in rhizosphere fungal communities. Numbers indicate the proportion of variation explained by the variable sets, and the overlap indicates that these changes cannot be explained by a single variable set. Monte Carlo analysis was used to examine the significance of the influence of variables on rhizosphere fungal community variation. *** *p* < 0.001; ** *p* < 0.01; * *p* < 0.05.

Using the db-RDA method, we observed the influence of two groups of variables: soil physicochemical properties and root traits (Figure 5a). The community structure was significantly influenced by the soil factors DOC, K, P, TC, and Ca, which had a high contribution rate in terms of soil physical and chemical properties. Moreover, while fungal community composition was significantly influenced by DOC, K, and P, RB, RC, and RD were found to be the primary factors driving changes in root fungal communities, highlighting their importance in shaping rhizosphere fungal communities. The responses of soil fungi to environmental factors differed at various developmental stages. Rhizosphere fungi in young and middle-aged forests showed positive responses to DOC and RB, while in over-mature forests they showed a positive response to P. Fungi in the 0–20 cm soil layer showed a positive response to RD, RC, and TC.

VPA showed that root traits and soil chemical properties are key factors affecting the variation in soil fungal communities in Chinese fir plantations. Together, these factors explain 26% of the variation in the fungal community (Figure 5b). Among them, the effect of soil physicochemical properties on fungal community structure (*p* < 0.001) accounts for 23% (but only 17% individually). The impact of root traits on fungal community structure (*p* < 0.001) accounts for 9% (with a single influence of 3%). In summary, the influence of soil physicochemical properties on the fungal community structure was much greater than that of the root traits.

### 3.5. Topological Characteristics of Co-Occurrence Networks of Rhizosphere Fungal Communities Across Developmental Stages in Chinese Fir Plantations

Topological analysis revealed clear stage-dependent changes in the rhizosphere fungal co-occurrence networks (Table S5). The number of nodes remained relatively stable across the four developmental stages, whereas the number of edges and average degree varied markedly. Network connectivity was the lowest in the networks for the middle-aged stands, which had the fewest edges and the lowest average degree, but was the highest in the over-mature stand networks, which had the highest number of edges and average degree. The over-mature stand networks also exhibited the shortest average path length and the highest clustering coefficient, indicating a more densely connected and locally clustered network. Closeness centralization also varied significantly among the developmental stages, whereas betweenness centralization and positive and negative cohesion showed no significant differences. Overall, these topological properties indicate a temporary reduction in network connectivity at the middle-aged stage, followed by increased connectivity toward the over-mature stage.

**Figure 6:**
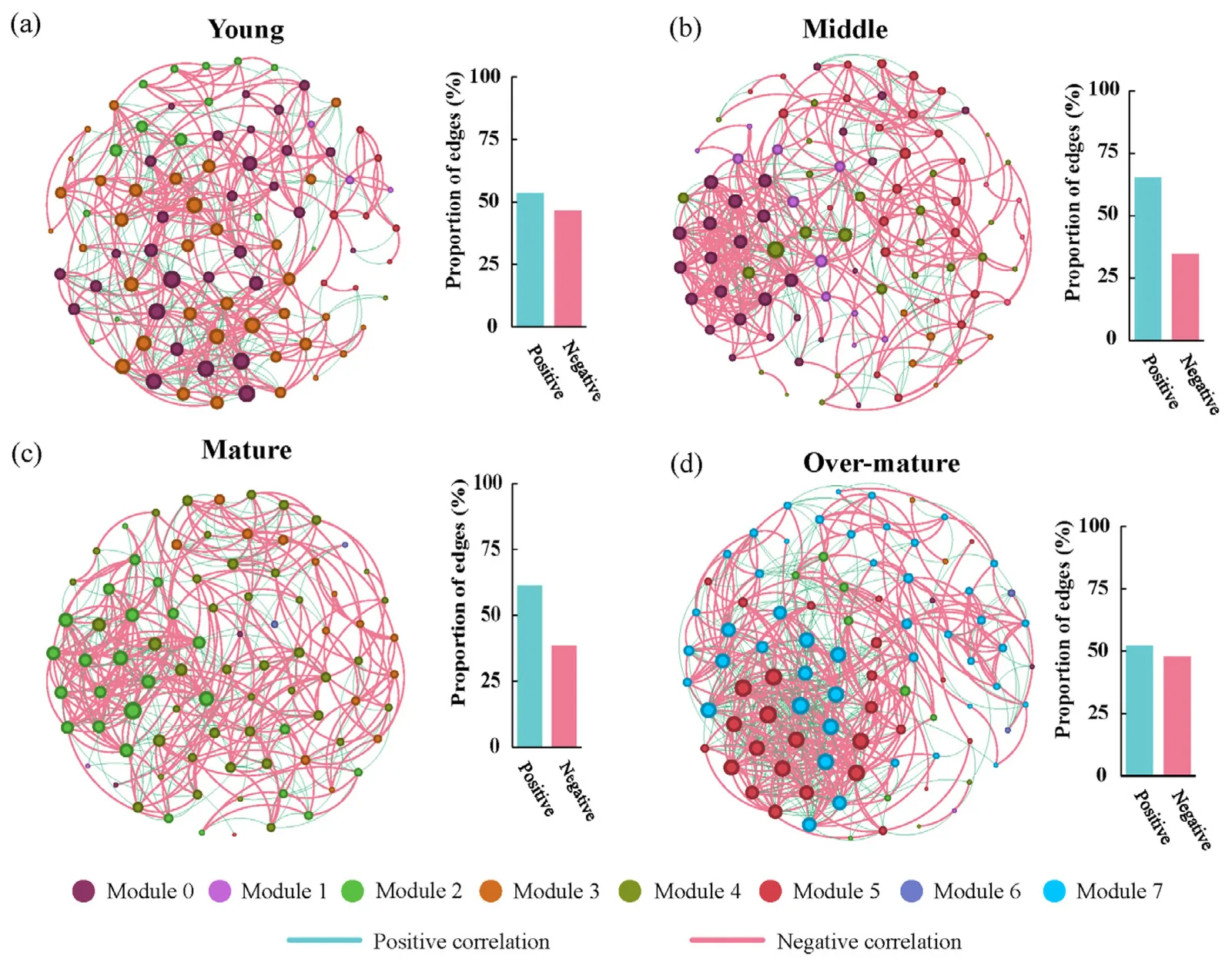
Co-occurrence networks of rhizosphere fungal communities across the different stand developmental stages of Chinese fir plantations. Each node in the network represents an OTU, and nodes are colored according to their network modules. (**a**) Young stand; (**b**) middle-aged stand; (**c**) mature stand; (**d**) over-mature stand.

Network visualization further revealed stage-dependent differences in the overall organization of fungal associations (Figure 6). Fungal taxa were organized into multiple modules at all developmental stages. Given the high density of network connections, Figure 6 illustrates the overall modular organization and relative proportions of positive and negative associations rather than individual pairwise or inter-module links. Positive associations predominated in all four networks; however, their relative proportions varied among developmental stages, with the highest proportion observed in middle-aged stands, followed by mature stands. By contrast, positive and negative associations were more balanced in young and over-mature stands. These patterns indicated that the overall organization and balance of fungal associations changed across stand developmental stages.

## 4. Discussion

### 4.1. Characteristics of Rhizosphere Fungi Biomass and Alpha-Diversity

The rhizosphere fungal biomass of Chinese fir was substantially influenced by the stand development stage, with young stands exhibiting significantly higher fungal biomass than the other three types of stands (Table 1). This pattern could be associated with higher root activity and greater rhizodeposition during the rapid growth stage of young plantations, which could provide more available carbon resources for rhizosphere fungi [30]. However, previous studies have shown that fungi in Chinese fir plantations are more sensitive to thinning than bacteria; thinning alters the rhizosphere fungal community composition, co-occurrence networks, and keystone taxa [31]. Moreover, thinning effects are not necessarily persistent; fungal diversity and community structure may recover after repeated thinning, particularly once the stand structure and soil nutrients have stabilized over time [32]. Therefore, the lower fungal biomass observed in older stands likely resulted from the combined effects of reduced root-derived carbon inputs, intensified inter-tree competition, and cumulative thinning disturbances rather than stand age alone. With the exception of fungal biomass, the other fungal indices in the soils of different ages did not change significantly, and the uniformity and diversity of the rhizosphere fungi remained relatively stable. This result is consistent with previous studies on the drivers of fungal communities in Chinese fir forests [11]. The relatively stable nutrient conditions in mature plantations may reduce environmental constraints on fungal communities, resulting in limited variation in fungal diversity [33].

With respect to soil depth, fungal biomass and α-diversity were generally higher in the 0–20 cm layer than in the 20–40 cm layer. This vertical distribution pattern is likely associated with the vertical heterogeneity of soil organic matter, nutrient availability, root activity, and microbial habitat conditions. Owing to the accumulation and decomposition of leaf litter, dead branches, and other plant residues, surface soil typically contains abundant organic matter (Table 1), which supports higher microbial biomass and provides energy for fungal growth [34]. Thinning may also influence this vertical pattern by modifying litter deposition, understory vegetation, and fine-root distribution; however, such effects are expected to be strongest in the upper soil layer and may diminish with increasing depth. Previous studies have consistently reported that microbial biomass and enzyme activities are generally higher in the rhizosphere and topsoil than in the subsoil, particularly in over-mature forests [35]. The functional importance of surface soil fungi is further reflected in their contribution to soil multifunctionality and nutrient cycling; key fungal groups involved in phosphorus transformation and soil organic carbon turnover have been reported to explain a substantial proportion of the variation in surface soil multifunctionality [33]. Collectively, these findings indicate that stand developmental stage, thinning history, and soil depth jointly regulate the rhizosphere fungal biomass and ecological functions in Chinese fir plantations.

### 4.2. Changes in Rhizosphere Fungal Community Structure and Composition

A multidimensional analysis revealed that as the four stand developmental stages progressed, the fungal community structure transitioned from aggregation to differentiation (Figure 1). This successional pattern should not be attributed solely to developmental stage, but rather interpreted within the context of contrasting management histories among the sampled stands. Accumulating evidence indicates that habitat modifications driven by forest management can outweigh age-related signals in shaping fungal community assembly [34,35,36,37]. Moreover, fungal communities exhibit pronounced sensitivity to gap-associated soil heterogeneity, with canopy gaps exerting a substantial influence on forest microenvironments [38]. Accordingly, the two thinning cycles experienced by mature and over-mature stands likely acted synergistically with stand development to promote the observed differentiation of rhizosphere fungal communities.

The prevalence of unidentified or unclassified fungal taxa in both the surface and deep soil layers was a conspicuous feature of community composition (Figure 2a). In this study, a relatively high proportion of fungal sequences (44.75%) could not be assigned to known genera. The multi-sample rarefaction curves gradually approached a plateau with increasing sequencing depth (Figure S2), indicating that the sequencing effort was sufficient to capture most of the detectable fungal diversity in the samples. This pattern may be attributable to the incomplete representation of fungal taxa in currently available reference databases and the limited taxonomic resolution of amplicon sequencing at the genus level. Previous studies have shown that fungal metabarcoding datasets frequently contain sequences that cannot be reliably assigned to meaningful taxonomic ranks, partly because many fungal lineages remain poorly characterized or are underrepresented in existing reference databases [39]. Therefore, whether these sequences represent novel fungal taxa remains to be determined. Future isolation, cultivation, and polyphasic taxonomic identification of these unclassified fungi would further deepen our understanding of belowground fungal diversity in Chinese fir plantations. The dominant phyla of soil fungi were consistently observed across the different developmental stages of Chinese fir plantations throughout the study. The most abundant dominant groups were Ascomycota and Basidiomycetes (Figure 2b). This pattern agrees with previous studies showing that these two phyla are dominant in forest soils [40,41,42,43]. The increasing abundance of Mortierellomycota and Chytridiomycota in mature and over-mature stands may be related to increased litter accumulation and organic matter inputs during forest development because members of these groups are often involved in organic matter decomposition [44,45].

Plant pathogens, wood saprotrophs, ericoid mycorrhizal fungi, and undefined saprotrophs were significantly affected during the different stages of stand development (Figure 3). With increasing forest age, in the abundances of plant pathogens and ericoid mycorrhiza declined. Compared with the other three stages, the mature stage showed significantly higher abundances of wood saprotrophs and undefined saprophytes (Table S3). As Chinese fir forests grow, the stand density and canopy increase, leading to decreased light and limited ventilation in the forest. This is not conducive to the spread and growth of pathogens [46]. The invasion of fungal pathogens seems to be more common during the early stages of Chinese fir forest development. In the mature stage, the accumulation of organic matter, such as dead branches and leaves, increased significantly, providing a rich source of nutrients that was conducive to the growth of wood saprotrophs and undefined saprotrophs. For example, many dead leaves are rich in cellulose, lignin and other components, and wood saprotrophs can effectively decompose these complex substances to obtain the energy and nutrients required for their growth [47]. Nevertheless, FUNGuild-based functional assignments should be interpreted with caution, because they rely on taxonomic information and reference databases. Some fungal taxa may exhibit multiple ecological strategies that cannot be fully captured by a single guild. Therefore, inferred changes in fungal guilds represent potential ecological shifts rather than direct evidence of functional changes [48].

Beyond the observed shifts in taxonomic composition, co-occurrence network analysis further demonstrated that stand development profoundly restructured the internal organization of the rhizosphere fungal communities (Table S4, Figure 6). Progressive increases in edge count, average degree, clustering coefficient, and closeness centralization across the young to over-mature stand gradient collectively indicate that forest maturation strengthens network connectivity and modularity. Elevated clustering coefficients (from 0.514 to 0.637) and closeness centralization (from 0.124 to 1.286) in the mature and over-mature stages indicated that fungal taxa formed tighter local associations and more distinct modular architectures, a pattern linked to enhanced functional differentiation and resource partitioning within microbial networks [49,50]. The emergence of core functional consortia within these modules likely reflects directional selection for specialized fungal clades adapted to the accumulated recalcitrant litter substrates characteristic of older stands [11,17]. Concurrently, a gradual increase in the proportion of positive edges and cohesion along the stand developmental gradient signals a shift from competitive to cooperative interspecific interactions. This pattern implies that niche differentiation among keystone fungal taxa diminishes with developmental stage, whereas facilitative relationships, such as those mediating the sequential degradation of complex organic matter, become increasingly dominant [51,52]. The transient decline in network connectivity at the middle-aged stage likely reflects the disruptive effects of thinning disturbances and intensified belowground competition during the rapid growth phase, which temporarily destabilize fungal co-occurrence patterns [13,22]. Overall, the transition toward more aggregated, functionally complex, and stable network architectures in older stands aligns with the consensus that mature forest ecosystems harbor more resilient and functionally integrated microbial communities [53]. These network-level findings complement the taxonomic compositional shifts documented above and provide mechanistic insights into how stand development shapes the assembly processes and functional organization of rhizosphere fungal communities in Chinese fir plantations.

### 4.3. Influence of Soil Physicochemical Properties and Root Traits on Rhizosphere Fungal Communities

Variance partitioning analysis was used to quantify the relative contributions of soil physicochemical properties and root traits to rhizosphere fungal community assembly. Collectively, these two groups of explanatory variables accounted for 26% of the total variation in rhizosphere fungal community structure, with edaphic properties independently explaining 17%, more than five times the 3% independent contribution of root traits. This quantitative partitioning established soil physicochemical properties, rather than root traits, as the dominant regulatory force shaping rhizosphere fungal community dynamics in Chinese fir plantations [7]. However, it is important to acknowledge that both soil properties and root traits are themselves substantially modified by forest management, particularly thinning. Previous studies have demonstrated that thinning can alter soil nutrient availability and functional root traits in Chinese fir plantations, with cascading effects on microbial communities [11,13]. Accordingly, the observed dominance of soil properties in structuring fungal communities likely reflects, in part, the contribution of stand thinning history.

Among the key edaphic variables, DOC exerted the strongest independent influence on fungal assembly, followed by K, P, and TC with distinct directional and stage-specific modes of action (Figure 5a). DOC dominated community structuring in young and middle-aged stands, where it positively supported plant pathogens and endophytic fungi through a labile carbon supply and improved soil aeration and water retention [51], but suppressed saprotrophic taxa, likely because of resource competition and niche differentiation. In contrast, K and P served as the primary drivers in mature and over-mature stands, predominantly promoting the growth of saprotrophic and arbuscular mycorrhizal fungi. The accumulation of plant litter in older stands enhances K and P availability, which in turn accelerates organic matter decomposition and increases carbon and nitrogen supply for saprotrophic growth [47,54,55]. Elevated soil phosphorus promotes symbiotic fungi such as arbuscular mycorrhizal fungi and ectomycorrhizal fungi by facilitating host plant phosphorus acquisition via mycelial networks [56,57,58]. TC primarily shaped surface (0–20 cm) fungal communities and exhibited positive associations with endophytic and lichenized fungi. Collectively, the regulatory hierarchy transitioned from carbon-dominated control in young stands to nutrient-dominated (K and P) control in older stands, thereby mediating the functional succession of rhizosphere fungal communities along the developmental gradient. Thinning could further modulate this transition by altering litter decomposition rates and nutrient release patterns.

Root traits also contributed to fungal community structure, albeit to a substantially lesser degree. RB, RD, and RC were identified as primary root-derived drivers (Figure 5a). RB was positively associated with rhizosphere fungi in young and middle-aged stands, which is consistent with the significantly higher RB and fungal richness observed in young stands (Table 1 and Table S2), as greater root biomass increases the quantity of rhizosphere deposits and root exudates that fuel microbial growth [59,60]. RD and RC primarily structured fungal communities in the 0–20 cm soil layer and were positively associated with ectomycorrhizal fungi, reflecting the link between root architectural traits and fungal symbiosis [61,62]. Notably, all saprotrophic fungal groups were negatively correlated with root traits (RB and RL), potentially because saprotrophic nutrient mineralization and plant root nutrient uptake are competing processes [55]. Collectively, variance partitioning analysis confirmed that soil physicochemical properties exerted a stronger influence on rhizosphere fungal community structure than root traits (Figure 5b), establishing edaphic conditions as the primary drivers of fungal assembly in Chinese fir plantations. Stand development acts as a pervasive ecological driver of soil microbial assembly across forest ecosystems and is underpinned by concurrent shifts in vegetation structure, resource availability, and edaphic properties during stand maturation. Although derived from subtropical Chinese fir plantations, our identified linkages between stand age, edaphic properties, root traits and fungal communities offer mechanistic insights into the belowground dynamics of other coniferous forests. Age-dependent fungal successional shifts have been widely documented [17,63], supporting stand ontogeny as a fundamental regulator of belowground microbial dynamics.

Nevertheless, fungal response patterns may vary with climate, baseline soil properties, host functional traits, and management regimes; therefore, the generalizability of our findings requires validation across broader regions and species. A key limitation is the absence of plot-scale quantitative in situ litterfall data. Age-driven shifts in litter quantity and quality likely partially underpin wood saprotroph enrichment and enhance cooperative interactions in older stands. Standardized long-term monitoring would help disentangle the independent role of litter in rhizosphere fungal assembly and successional trajectories. Future studies should explicitly incorporate thinning history as a controlled treatment in experimental designs, thereby enabling the separation of the direct effects of stand development from the indirect effects of silvicultural interventions.

## 5. Conclusions

This study delineated the successional patterns and driving forces of rhizosphere soil fungal communities across the developmental stages of Chinese fir plantations. Along the developmental stage gradient, fungal biomass peaked at the young stage while α-diversity remained stable, and community structure shifted progressively from an aggregated to a divergent state. Ascomycota and Basidiomycota persistently dominated all stages, whereas the low-abundance Mortierellomycota and Chytridiomycota exhibited strong age-dependent sensitivities. Co-occurrence networks showed increasing connectivity and modularity along succession, with a shift from competitive to cooperative interspecific interactions and a transient connectivity decline at the middle-aged stage.

Variance partitioning analysis confirmed that soil physicochemical properties were the dominant regulators, independently explaining 17% of the total community variation, over five times the 3% independent contribution of root traits. DOC exerted the strongest edaphic effect, followed by K, P, and TC. DOC positively supported pathogenic and endophytic fungi but suppressed saprotrophic taxa, dominating in young and middle-aged stands; K and P promoted saprotrophic and mycorrhizal fungi and prevailed in mature and over-mature stands. This carbon-to-mineral-nutrient regulatory shift underpins fungal functional succession.

These findings suggest that soil management in Chinese fir plantations should be tailored to stand developmental stages, with greater attention paid to soil carbon availability in young and middle-aged plantations and to K and P status in mature and over-mature plantations, thereby supporting favorable rhizosphere fungal functions and the long-term sustainability of Chinese fir plantations.

## Figures and Tables

**Table 1:** Copies and α-diversity of rhizosphere fungus in Chinese fir plantations at different stand developmental stages (mean ± SE).

SDS	D (cm)	Copies (×10^5^ Copies·g^−1^)	Chao1 Richness Index	Observed Species	PD Whole Tree Index	Shannon-Wiener Diversity Index
Young	0–20	69.23 ± 46.71 a	745.29 ± 32.93 a	506.17 ± 37.62 a	99.34 ± 4.57 a	6.21 ± 0.41
	20–40	43.67 ± 25.92 a	675.30 ± 61.55 a	445.73 ± 40.02 a	88.05 ± 6.59 a	6.44 ± 0.12
Middle-aged	0–20	1.10 ± 0.54 bB	734.80 ± 21.25 A	484.57 ± 14.41 a	96.81 ± 1.73 aA	6.42 ± 0.22
	20–40	4.28 ± 0.82 bA	573.83 ± 47.31 aB	407.87 ± 26.44 a	83.66 ± 3.56 aB	5.94 ± 0.34
Mature	0–20	8.53 ± 3.58 bA	684.45 ± 39.06 a	503.90 ± 31.02 aA	104.32 ± 6.61 a	6.61 ± 0.19
	20–40	1.73 ± 0.69 bB	528.61 ± 50.85 a	382.27 ± 40.36 aB	84.40 ± 6.74 a	5.71 ± 0.43
Over-mature	0–20	6.83 ± 0.25 bB	649.07 ± 45.75 a	458.87 ± 16.85 a	92.74 ± 4.85 a	6.31 ± 0.24
	20–40	0.67 ± 0.16 bA	511.27 ± 85.74 a	381.63 ± 52.11 a	82.15 ± 9.62 a	6.29 ± 0.28
Two-way ANOVA						
SDS		0.030 *	0.098	0.473	0.636	0.922
D		0.519	0.002 **	0.003 **	0.005 **	0.178
SDS × D		0.893	0.801	0.832	0.856	0.277

Stand developmental stage, SDS; Soil depth, D. Different lowercase letters within the same column indicate significant differences in stand developmental stages within the same soil layer at *p* < 0.05. Different capital letters in the same column indicat significant differences across soil layers at the same developmental stage at *p* < 0.05. **, *p* < 0.01; *, *p* < 0.05.

## Data Availability

The original data presented in the study are openly available in NCBI with accession number PRJNA1520018.

## Supplementary Materials

The following supporting information can be downloaded at: https://www.mdpi.com/article/10.3390/microorganisms14092078/s1, Figure S1: Venn diagrams of internal transcribed spacer DNA for high-throughput sequencing of fungi of stand development at two different soil depths ((a) 0–20 cm and (b) 20–40 cm) in Chinese fir plantations. Each data point in the graph represents the fungal count at two soil depths and four stages of stand development, and the percentage in parentheses represents the percentage of fungal count at the two soil depths and four stages of stand development; Figure S2: Rarefaction curves of OTUs for each sample across different stand development stages (at 97% similarity level); Table S1: Soil physical and chemical properties in Chinese fir plantations with different stand developmental stages; Table S2: The traits of tree roots in Chinese fir plantations with different stand developmental stages; Table S3: Relative abundance of the most abundant fungal groups in different developmental stages of Chinese fir stands at the phylum level; Table S4: The relative percentage of FUNGuild classifications at the genus level based on the fungal ecological functional roles among different developmental stages of Chinese fir plantations at depths of 0–20 cm and 20–40 cm; Table S5: Topological properties of fungal co-occurrence networks in the rhizosphere soil across different developmental stages of Chinese fir plantations.
