# Supplementary material for: Rhizosphere Soil Fungal Community Patterns and Drivers in Chinese Fir Plantations Across Developmental Stages
Source: Microorganisms. 2026 Sep 17;14(9):2078. doi: 10.3390/microorganisms14092078 (PMC13609283; doi:10.3390/microorganisms14092078)
Supplement: Supplementary file 1 [file microorganisms-14-02078-s001.zip › microorganisms-4512214-supplementary.pdf]

Table S1. Soil physical and chemical properties in Chinese fir plantations with different stand developmental stages.

Stand Development Stage	Depth D (cm)	WC (%)	pH	TC ($g·kg^{-1}$)	TN ($g·kg^{-1}$)	TP ($g·kg^{-1}$)	AN ($mg·kg^{-1}$)	NN ($mg·kg^{-1}$)	DOC ($mg·kg^{-1}$)	AP ($mg·kg^{-1}$)	Mg ($g·kg^{-1}$)	K ($g·kg^{-1}$)	Ca ($g·kg^{-1}$)
Young-aged stand	0-20	23.47±1.37	4.09±0.04	28.35±0.49A	1.88±0.03A	0.14±0.03A	11.38±3.12	7.98±3.15	1276.16±219.87a	6.67±1.45ab	1.45±0.10b	16.32±0.95c	0.24±0.05b
	20-40	23.16±1.34	4.17±0.07	21.81±2.15aB	1.01±0.13aB	0.10±0.02B	8.47±1.43	4.37±1.34	1217.24±138.54a	3.90±0.12	1.52±0.10b	18.92±0.28b	0.23±0.05b
Middle-aged stand	0-20	24.04±1.08A	3.97±0.06	30.65±2.06A	1.59±0.22A	0.12±0.01A	13.09±5.78	11.62±4.77	988.06±30.25ab	4.42±0.89b	2.89±0.11a	25.37±0.82b	0.48±0.02a
	20-40	20.01±0.16B	4.07±0.06	16.70±1.89abB	0.72±0.08ab	0.10±0.01B	5.41±0.41	8.28±3.12	958.17±41.42ab	3.58±0.53	2.92±0.10a	28.04±2.08b	0.38±0.07ab
Mature stand	0-20	22.23±1.43	4.17±0.04	22.89±1.73A	1.25±0.28	0.14±0.01A	11.37±0.19A	13.61±2.79A	504.16±51.76c	3.84±0.10bA	2.85±0.19a	46.84±5.34a	0.32±0.06b
	20-40	19.31±1.46	4.09±0.02	13.13±0.88bB	0.62±0.04bB	0.11±0.00B	6.99±0.43B	5.81±2.04B	582.38±62.51c	2.21±0.41B	2.95±0.19a	45.21±5.63a	0.16±0.07b
Over-mature stand	0-20	24.26±3.23	3.95±0.05B	31.28±5.45A	2.02±0.58A	0.19±0.02	13.38±2.40	15.39±7.93	737.38±88.75bc	11.10±3.37a	2.46±0.21a	23.81±0.74c	0.55±0.03a
	20-40	16.51±1.76	4.30±0.06A	13.32±1.60abB	0.63±0.05bB	0.14±0.04	8.30±2.11	4.70±2.02	564.71±146.07bc	6.29±2.74	2.66±0.03a	26.46±1.72b	0.57±0.12a
ANOVA													
SDS	0.324	0.151	0.024*	0.229	0.116	0.915	0.718	<0.001***	0.019	<0.001***	<0.001***	<0.001***	0.324
D	0.006**	0.007**	<0.001***	<0.001***	0.040*	0.016*	0.036*	0.581	0.049*	0.327	0.462	0.194	0.006**
SDS×D	0.208	0.006**	0.118	0.485	0.886	0.835	0.750	0.754	0.664	0.941	0.852	0.488	0.208

Stand developmental stage, SDS; Soil depth, D. Different lowercase letters within the same column indicated a significant difference in stand development stages within the same soil layer at the level of $P < 0.05$. Different capital letters in the same column indicated a significant difference across different soil layers at the same development stage at the level of $P < 0.05$. ***, $P < 0.001$; **, $P < 0.01$; *, $P < 0.05$.

Table S2. The traits of tree roots in Chinese fir plantations with different stand developmental stages.

Stand Development Stage	Root Biomass ($g·m^{-2}$)	Root Length ($m·m^{-2}$)	Root Surface Area ($m^2·m^{-2}$)	Root Diameter (cm)	Specific Root Length ($m·g^{-1}$)	Specific Surface Area ($cm^2·g^{-1}$)	Root C Content ($mg·g^{-1}$)	Root N Content ($mg\ g^{-1}$)	Root P Content ($mg\ g^{-1}$)
0-20 cm									
Young	84.42±30.19a	250.35±110.94a	0.72±0.28a	0.96±0.10B	2.92±0.50abA	84.91±7.16A	461.78±8.33	9.39±0.67	0.38±0.02
Middle	59.63±24.19ab	93.31±31.74ab	0.34±0.11ab	1.20±0.14	1.68±0.35b	60.33±6.82	457.25±1.50	8.73±0.28	0.38±0.06
Mature	18.44±5.43b	61.55±3.39b	0.17±0.01b	0.88±0.09	3.91±1.00a	101.42±17.69	450.57±3.21	8.53±0.54	0.43±0.05
Overmature	41.18±13.42ab	98.56±15.45abA	0.31±0.08ab	0.95±0.11	2.71±0.49abA	78.35±7.04	458.44±9.50	9.65±0.26A	0.32±0.03
20-40 cm									
Young	60.08±15.21a	100.35±25.82a	0.38±0.09a	1.24±0.06A	1.67±0.10abB	64.36±2.54B	464.59±9.18	6.43±1.03b	0.30±0.01

Middle	30.32±11.50ab	41.33±15.41ab	0.18±0.06ab	1.42±0.19	1.51±0.35b	63.57±7.88	450.68±5.62	9.24±0.36a	0.33±0.04
Mature	27.35±15.54ab	57.52±29.23ab	0.18±0.10ab	0.97±0.08	2.25±0.18a	68.00±3.40	446.93±7.01	7.97±0.70ab	0.30±0.04
Overmature	21.61±10.66b	25.19±6.94bB	0.12±0.05b	1.38±0.20	1.42±0.26bB	60.27±5.51	444.83±14.29	8.88±0.12aB	0.34±0.07
SDS	**0.035**	**0.014**	**0.015**	0.063	**0.045**	0.090	0.359	0.101	0.847
D	0.176	**0.002**	**0.019**	**0.014**	**0.005**	**0.011**	0.380	**0.032**	0.059
SDS×D	0.633	0.430	0.568	0.633	0.464	0.224	0.795	**0.049**	0.356

Data were expressed as mean ± standard deviation SD; Different lowercase letters within the same column indicated a significant difference in stand development stages within the same soil layer at the level of $p < 0.05$. Different capital letters in the same column indicated a significant difference across different soil layers at the same development stage at the level of $p < 0.05$.

Table S3. Relative abundance of the most abundant fungal groups in different developmental stages of Chinese fir stands at the phylum level.

**Stand Developmental Stage**	**Ascomycota**	**Basidiomycota**	**Chytridiomycota**	**Unidentified**	**Glomeromycota**	**Rozellomycota**
0-20cm						
Young	55.83±8.45	34.64±9.12	0.63±0.14	4.04±0.23	0.26±0.04	3.31±1.45
Middle	73.42±3.62	20.08±3.39	0.40±0.11	2.21±0.53	0.69±0.23	1.84±0.93
Mature	54.76±3.38	25.40±3.53	7.89±5.20	2.58±0.37	0.56±0.07	3.04±1.74
Overmature	63.28±6.95	23.08 ±3.76	4.23±3.77	6.00±0.46	0.73±0.30	1.17±0.64
20-40cm						
Young	66.61±2.73	24.68±3.95	0.38±0.15	3.07±0.63	1.24±0.66	2.66±1.62
Middle	70.05±4.66	22.95±5.15	0.43±0.19	2.75±0.28	0.39±0.10	1.78±1.34
Mature	65.55±1.72	23.11±1.18	3.60±2.75	3.03±0.37	0.37±0.08	1.45±0.97
Overmature	65.36±3.61	24.21±3.67	0.73±0.06	5.12±0.48	1.69±0.65	1.31±0.62
Two-way ANOVA						
SDS	0.116	0.387	0.000	0.197	0.197	0.557
D	0.160	0.546	0.500	0.172	0.172	0.543
SDS*D	0.408	0.552	0.197	0.164	0.164	0.896

Data are presented as the means of three replicates with SE (n = 3). Different lowercase letters within a column indicate significant differences among the stand developmental stages in the same soil layer ($p < 0.05$). The P values in bold indicate statistically significant differences among SDS, D) or their interaction determined using two-way ANOVA ($p < 0.05$).

Table S4. The relative percentage of FUNGuild classifications at the genus level based on the fungal ecological functional roles among different developmental stages of Chinese fir plantations at depths of 0-20 cm and 20-40 cm.

**Stand Developmental Stage**	**Plant Pathogen**	**Animal Pathogen**	**Endophyte**	**Ectomycorrhizal**	**Ericoid Mycorrhizal**	**Fungal Parasite**	**Dung Saprotroph**	**Litter Saprotroph**	**Wood Saprotroph**	**Undefined Saprotroph**	**Undefined**
					**0-20 cm**						
Young-aged	12.20±2.96AB	0.37±0.03	3.57±0.95	1.75±0.79	0.99±0.24	0.09±0.04	1.15±0.25	0.14±0.07	0.62±0.23	2.41±0.69B	76.67±5.87

Middle-aged	21.57±5.10 A	0.10±0.03	3.57±0.87	2.79±1.18	0.95±0.36	0.03±0.01	1.87±0.76	0.07±0.03	1.97±0.42	3.50±0.78 B	63.37±2.45
Mature	5.44±0.98B	0.68±0.08	6.15±1.65	1.40±0.21	0.60±0.11	0.46±0.04	2.49±0.95	0.77±0.22	4.02±2.28	6.19±0.85 A	71.62±2.34
Overmature	3.35±0.64B	0.99±0.71	9.68±6.11	0.97±0.27	0.32±0.16	0.14±0.06	2.14±0.18	0.37±0.27	0.43±0.16	2.05±0.27 B	79.44±5.00
**20-40 cm**											
Young-aged	11.56±2.31 A	0.32±0.14	2.20±0.40	2.75±1.00	1.58±0.40 A	0.10±0.07	1.91±1.15	0.11±0.05	0.73±0.34 B	2.47±0.41	76.16±3.93
Middle-aged	14.14±4.56 A	0.13±0.05	3.93±2.00	2.93±0.59	0.85±0.47 AB	0.07±0.03	1.75±0.24	0.11±0.02	0.79±0.48 B	2.20±0.23	72.92±5.67
Mature	2.22±0.18B	0.62±0.27	1.83±0.31	1.72±0.63	0.27±0.10 B	0.16±0.05	1.72±0.48	0.10±0.08	8.62±1.09 A	2.75±0.56	79.86±0.11
Overmature	2.11±0.52B	0.20±0.05	2.68±1.30	1.42±0.87	0.26±0.15 B	0.39±0.29	4.66±1.38	0.23±0.17	0.33±0.11 B	1.73±0.05	85.45±1.15
**Two-way ANONA**											
SDS	**<0.001**	0.223	0.589	0.172	**0.010**	0.084	0.135	0.098	**<0.001**	**0.002**	**0.017**
D	0.133	0.275	0.053	0.387	0.907	0.986	0.303	0.068	0.212	**0.005**	0.218
SDS×D	0.622	0.433	0.469	0.948	0.426	0.136	0.230	0.096	**0.032**	**0.025**	0.585

The numbers in parentheses indicate the SE (n=3). The *P* values in bold indicate statistically significant differences among SDS, D or their interaction identified using two-way ANOVA (p<0.05). Different letters indicate significant differences among stand developmental stages at the same soil depth (p<0.05).

Table S5. Topological properties of fungal co-occurrence networks in the rhizosphere soil across different developmental stages of Chinese fir plantations.

Network Parameter	Stand Developmental Stage			
	Young	Middle	Mature	Over-Mature
umber of nodes	58±3a	56±3a	56±2a	56±2a
Number of edges	173±22b	132±17b	179±17b	237±19a
Average degree	5.827±0.420bc	4.689±0.515c	6.396±0.461b	8.376±0.419a
Average path length	3.054±0.116b	3.578±0.109a	2.867±0.140b	2.492±0.112c
Clustering coefficient	0.514±0.013b	0.580±0.036ab	0.579±0.025ab	0.637±0.016a
Closeness centralization	0.124±0.009c	1.567±0.139a	1.147±0.049b	1.286±0.110b
Betweenness centralization	0.192±0.033a	0.213±0.019a	0.164±0.021a	0.135±0.023a
Positive cohesion	0.658±0.016a	0.673±0.012a	0.663±0.027a	0.624±0.018a
Negative cohesion	-0.577±0.028a	-0.566±0.012a	-0.623±0.039a	-0.610±0.024a

Young,Young-aged stand; Middle, Middle-aged stand; Mature, Mature stand; Over-mature, Over-mature stand.

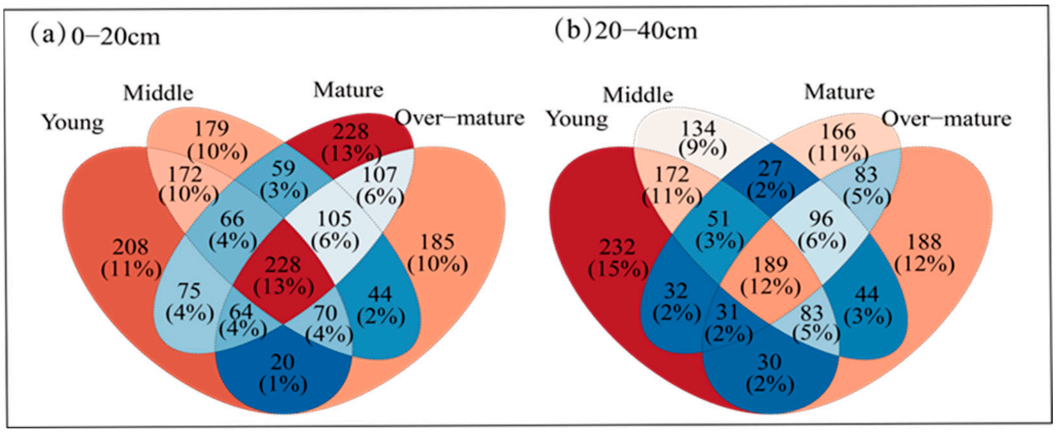

Figure S1. Venn diagrams of internal transcribed spacer DNA for high-throughput sequencing of fungi of stand development at two different soil depths (（a）0-20 cm and (b) 20-40 cm) in Chinese fir plantations. Each data point in the graph represents the fungal count at two soil depths and four stages of stand development, and the percentage in parentheses represents the percentage of fungal count at the two soil depths and four stages of stand development.

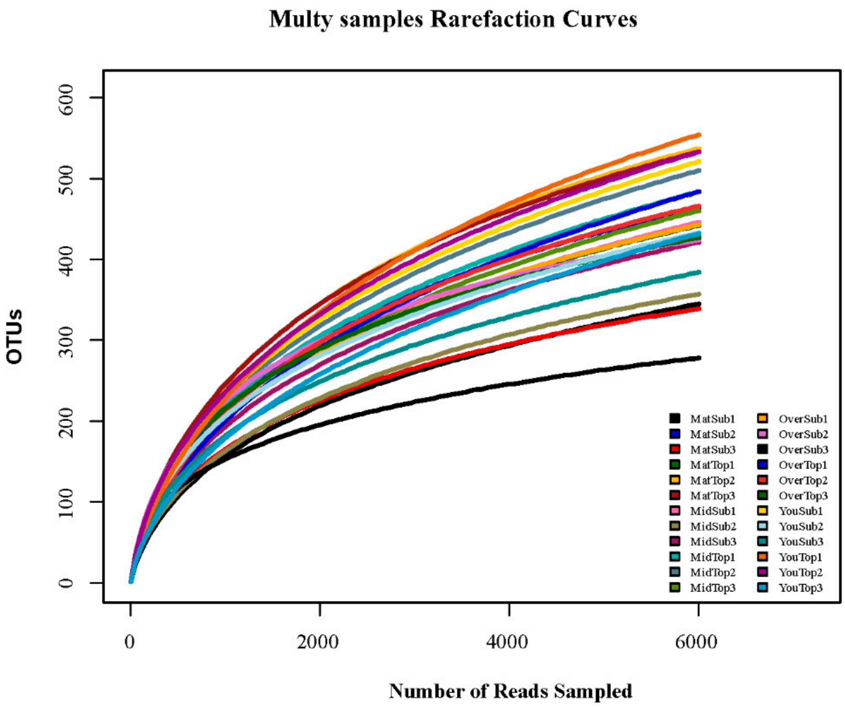

Figure S2. Rarefaction curves of OTUs for each sample across different stand development stages (at 97% similarity level).
